# Supplementary material for: Filling the GAP: Integrating a gambling addiction program into a shelter setting for people experiencing poverty and homelessness
Source: PLoS One. 2022 Mar 15;17(3):e0264922. doi: 10.1371/journal.pone.0264922 (PMC8923431; doi:10.1371/journal.pone.0264922)
Supplement: S1 File — (PDF) [file pone.0264922.s001.pdf]

Appendix 18

Good Shepherd Ministries Poverty Reduction Program
Interview Questions

Intervention (Phase II): Interview

[Please read to participant]

Thank you for agreeing to participate in this interview. We appreciate the time you are taking to speak with us today. The interview should last about 60 minutes.

I'm going to read each question for you and at times will ask you to provide more detail or ask additional questions based on your responses. Please let me know at any time if the meaning of the question is unclear and I will ask it in another way. I'd like you to answer in your own words. Please remember, there are no "right or wrong" answers. We are really interested in your experiences so all of the information you provide is important. Also you can choose to not answer or "pass" on answering any question, or stop the interview at any time.

Do you have any questions or comments before we start recording?

[Wait for response and respond to any questions]

I'm going to turn on the recorder now and will let you know when we stop recording.

[Turn on recorder]

This is [interviewer's name] and it is [date] and I am here with [participant ID] at [interview location] and we are about to start the interview.

Thank you again for agreeing to talk with me today about your thoughts and experiences with gambling. As someone with this experience, our team highly values what you have to say, so thank you.

1. So to begin, what do you think about us doing this research on ways to help people manage their gambling? (Probe: Do you think is it important that we talk to people like yourself who have first-hand experience with gambling?)

I am hoping to learn more about you, your housing and gambling activity.

2. Could you please tell me about your housing situation right now? (Probe: housing type; living alone or with other people)
3. How has your housing situation changed since you met with a gambling addiction case worker and joined the program, if at all?
    a. How did accessing GSM's services contribute in stabilizing your housing situation, if at all?
4. How have your experiences in the program affected your gambling? (Probe: Frequency, money spent)
5. How do you think gambling has affected your housing? (Probe: Has gambling affected your finances at all?)

I am hoping to learn more about your experiences and thoughts regarding the new Gambling Addiction program at GSM so that we can find ways of making it better for clients.

6. Are you currently enrolled or were enrolled at a certain point into the gambling addiction program?
    a. **If yes, continue.**
    b. **If no, go to question 36.** (**Instruction for Interviewer**: *if client answers no it means they decided to be enrolled into the research but did not participate in the gambling addiction program.)*
7. How did you find out about the program at GSM?
8. What do you think would be the best way to let people know about this program?
9. What kind of support did or are you choosing to access as part of the gambling addiction program? (Case management; CBT; Life Skills; Gambler Anonymous)

(**Instruction for Interviewer**: *Based on the answer go to question 10, 15, 20 or 25 to learn more about client's experience with each part of the program they might have attended)*

Case Management

I would like to learn more about your experiences in case management with your gambling addiction case worker

10. How often did you meet?
11. What did you discuss or do during the sessions? (Probe: Make plans, address other needs)

12. Was it helpful to meet one-on-one, or would group sessions be just as helpful? (Probe: More confidentiality, comfort)
13. How did case management affect your PG, if at all? (Probe: Help reduce PG harms? If so, how?)
14. What else could be changed to make case management more helpful for you? (Probe: More/fewer meetings, discuss different topics, more resources)

Groups

I would like to learn more about your experiences with the **Cognitive Behavioural Therapy group (CBT)**.

15. How many sessions did you attend, if any? (Probe: If low attendance rate, ask what were the reasons for attending part of the sessions)
16. What did you like best about the group? (Probe: What worked well?)
17. What information did you find most useful from the group? (Probe: the most important thing you learned; how did you use what you learned to manage your gambling)
18. What did you like least about the group? (Probe: What did not work well?)
19. What do you think could be changed about the group to make it better? (Probe: Scheduling, topics discussed, format)

I would like to learn more about your experiences with the **Life Skill group.**

20. How many sessions did you attend, if any? (Probe: If low attendance rate, ask what were the reasons for attending part of the session)
21. What did you like best about the group? (Probe: What worked well?)
22. What information did you find most useful from the group? (Probe: the most important thing you learned; how did you use what you learned to manage your gambling)
23. What did you like least about the group? (Probe: What did not work well?)
24. What do you think could be changed about the group to make it better? (Probe: Scheduling, topics discussed, format)

I would like to learn more about your experiences with the **Gambler Anonymous group**

25. How many sessions did you attend, if any? (Probe: If low attendance rate, ask what were the reasons for attending part of the session)

26. What did you like best about the group? (Probe: What worked well?)
27. What information did you find most useful from the group? (Probe: the most important thing you learned; how did you use what you learned to manage your gambling)
28. What did you like least about the group? (Probe: What did not work well?)
29. What do you think could be changed about the group to make it better? (Probe: Scheduling, topics discussed, format)

I am wondering what you learned about yourself from participating in this Gambling Addiction Program.

30. More specifically, what did you learn about your gambling behaviour? (Probe: triggers strategies to self-manage gambling; negative consequences of gambling)
    a. Do you feel more in control of your gambling activity? If so, what has helped you manage it? If not, what might help you manage it better?
31. Has the Gambling Addiction Program helped you?
    a) If so in what ways?
    b) If not, what part of the program could have been better?
32. How has the way you thought about your gambling changed since participating in the program? (Probe: Do you open up and talk with others about your problem?)

We are interested in knowing which other services you do or would like to access at GSM.

33. Did your case worker refer you to any other programs within GSM or outside? (Probe: DARE; Financial Counselling; Volunteer Trusteeship; Eviction Prevention; Residential Resettlement)
    a) Could you tell me more about your experience with the specific programs?
34. What additional supports or services might you need in addition to what is offered at GSM?

(**Instruction for Interviewer**: *this section specifically addresses those who interrupted their attendance in one or more groups of the program. You will go through this if the interviewee mentions suspending their attendance in previous answers*)

<u>Service interrupted:</u> *I am interested in learning why you decided not to continue participating in the Gambling Addiction program.*

35. Are there any particular reasons why you decided to interrupt your participation into the program? (Probe: Reasons related to personal life events/crisis; dissatisfaction with the program; different expectations)
    a) Do you plan to go back into the program in the future? Why or why not.

(**Instruction for Interviewer**: *Skip this section and proceed to final question if the client took part in at least one part of the Gambling Addiction program)*

Control Group*: I am interested in understanding why you decided not to take part into the Gambling Addiction Program.*

36. What made you decide not to take part in the program? (Probe: Other needs/ life circumstances? Could the program have been introduced to you in a different better way?)
37. Have you heard anything about the program from other people? (Probe: What have you heard?)
38. What would make you more interested in participating in the gambling addiction program?
39. What services and supports are you receiving at GSM? Are you involved in any groups or programs? (Probe: Residential Resettlement; DARE; Financial Counselling; Volunteer Trusteeship; Eviction Prevention; Residential Resettlement)?
    a) Have any of these programs changed the way you gamble? (Probe: Have they helped you better manage your gambling? Which program and how?)

Final question.

40. Is there anything else you wanted to add or is there something I should have asked, but didn't?

Closing statements/ questions:

We talked about many things today. Thank you for sharing these things with me. As this comes to an end, is there anything you would like to add? Is there something I didn't ask but should have based on your experiences?

[Pause for response. Address any topics raised by the participant.]

I'll turn off the recorder now. Now that the recorder is off do you have any questions or comments you'd like to talk about off the record?

[Address any comments or questions as needed.]

Thank you again for your time and reflections.
